# The Complexity of Mental Health App Privacy Policies: A Potential Barrier to Privacy

**DOI:** 10.2196/mhealth.9871

**Published:** 2018-07-30

**Authors:** Adam C Powell, Preeti Singh, John Torous

**Affiliations:** ^1^ Payer+Provider Syndicate Boston, MA United States; ^2^ Indian School of Business Sahibzada Ajit Singh Nagar India; ^3^ Max Institute of Healthcare Management Indian School of Business Sahibzada Ajit Singh Nagar India; ^4^ Department of Psychiatry Beth Israel Deaconess Medical Center Harvard Medical School Boston, MA United States

**Keywords:** apps, privacy, ethics, mobile phone

## Abstract

**Background:**

In 2017, the Supreme Court of India ruled that privacy is a fundamental right of every citizen. Although mobile phone apps have the potential to help people with noncommunicable diseases, such as diabetes and mental illness, they often contain complex privacy policies, which consumers may not understand. This complexity may impede the ability of consumers to make decisions regarding privacy, a critical issue due to the stigma of mental illness.

**Objective:**

Our objective is to determine whether mental health apps have more complex privacy policies than diabetes apps.

**Methods:**

The study used privacy policies extracted from apps. The apps pertained to diabetes or mental health, and were all of Indian origin. Privacy policy reading complexity was compared between the two types of apps using a series of 15 readability measures. The universe of applicable apps on the Google Play store, as viewed between May and June 2017, was considered. The measures of readability were compared using chi-square tests.

**Results:**

No significant difference was found between the privacy policy readability of the diabetes apps versus the mental health apps for each of the measures considered. The mean Flesch-Kincaid Grade Level was 13.9 for diabetes apps and 13.6 for mental health apps; therefore, the mean policy grade level for both types of apps was written at a college level. Privacy policies in the 25th percentile of complexity were also written at a college level for both types of apps.

**Conclusions:**

Privacy policy complexity may be a barrier for informed decision making.

## Introduction

The Supreme Court of India’s August 2017 ruling that privacy is a fundamental right of every citizen underscores the need for greater attention to privacy rights in all contexts of Indian society [1]. Indians’ rights to privacy are only truly protected if Indians are able to make conscious decisions about their privacy-related decisions in all contexts, including while surrendering rights in the process of agreeing to privacy policies. The issue of privacy is especially important for mHealth apps, which are showing strong potential for addressing noncommunicable disease in developing nations such as India [2]. Although more than 40% of Americans believe that mental illness is similar to physical illness, less than 20% of Indians agree with this sentiment [3]. Due to this attitudinal difference, it is possible that there is a greater distinction between the privacy policies of apps for mental health versus physical health in India than in the United States.

Although privacy is an important issue for all users of mHealth apps, regardless of condition or location, in 2013, India lost more than 30 million disability-adjusted life years (DALYs) to mental, neurological, and substance abuse disorders—a 61% increase over the quantity in 1990. By comparison, all developed countries combined lost 50 million DALYs [4]. Noncommunicable physical illnesses are also afflicting a substantial number of Indians. India has been named the diabetes capital of the world [5]. It had a population of more than 72 million citizens with diabetes in 2017 and is projected to have 151 million citizens with diabetes in 2045 [6]. Given the substantial and growing number of people experiencing mental illness and diabetes within India and the greater degree of stigma associated with mental illness in India than in the United States [3], the potential for users of both physical and mental health apps to make informed privacy decisions is important to assess. The recent declaration of a fundamental right to privacy in India has amplified the importance of assessing the potential difficulty that users of Indian mHealth apps may have while attempting to preserve their privacy.

Recent research has raised concerns that irrespective of health benefits, there are inadequate privacy protections within mobile phone apps. One study examining the privacy policies of 211 diabetes apps noted that of those apps investigated, 81% did not even offer a privacy policy [7]. Another study attempting to examine the privacy policies of 72 dementia apps found that more than 50% lacked a privacy policy [8]. Privacy policies are of central importance because the majority of health apps live outside of the jurisdiction of national or federal health care regulations, meaning that privacy of information collected by a mobile phone app is not guaranteed in the same way as information shared with a doctor [9]. The US Department of Health and Human Services acknowledged the scope of this problem in a recent report outlining the extent to which consumers may be unaware of what data they are disclosing when using health-related mobile phone apps, who is able to access their data, and how their data may be sold or bartered [10]. The recent misuse of personal data, including personality profiles, in the 2018 Facebook and Cambridge Analytica scandal highlights the potential magnitude of harm resulting from inappropriate access and the global nature of such risks [11]. Thus, health app privacy policies that consumers can access and understand are necessary for consumers to protect their privacy rights and control what happens to their personal data.

Even when a privacy policy is present, it may not be comprehended by vulnerable consumers, such as those with mental illnesses that impair cognition [12,13]. Prior studies have characterized app privacy policies as being lengthy, linguistically complex, and even absent [14,15]. They have been shown to be difficult to read even for people pursuing a graduate degree in law or policy [16]. Although it is already well established that online privacy policies are challenging to read [17-20], the problem is even more pronounced within apps; it has been demonstrated that privacy policies are more difficult to read on a mobile device than on a desktop [21]. One study suggested that those with lower health literacy might misjudge privacy policies and falsely assume more protections are in place than those who are more health literate [14]. However, little research has been done to examine the reading level required to understand mobile health app privacy policies. No studies have examined whether the complexity of privacy policies differs according to the condition apps are intended to address. Furthermore, although much of the prior research on privacy policies has been conducted in the United States, there are national differences in privacy concerns, which reflect both differences in culture and values [22]. Thus, in this study we sought to characterize and compare the reading level of privacy policies for Indian apps intended to address issues related to diabetes and mental health.

## Methods

### Study Design and Data Sources

This study examined the complexity of privacy policies found within Indian apps for issues related to diabetes and mental health. The study used a novel dataset composed of privacy policies extracted from apps found on the Google Play app store for the Android operating system between May and June 2017 by a researcher based in India. Institutional Review Board approval was unnecessary because the subject of the research was software rather than people.

### Sample Selection

The Google Play store was searched for Indian apps related to diabetes and mental health. The apps returned by queries for “Indian diabetes,” “Indian diabetic,” or “Indian diabetes help” were included in the set of diabetes apps. The apps returned by queries for “Indian mental health,” “Indian anxiety,” “Indian depression,” “Indian schizophrenia,” “Indian posttraumatic stress disorder,” “Indian mood disorder,” “Indian cognitive behavior therapy,” “Indian cognitive remediation,” “Indian dialectical behavior therapy,” “Indian dementia,” or “Indian Alzheimer” were included in the set of mental health apps. Apps were excluded from the analysis if they were unrelated to health, despite containing health-related keywords, if they contained keywords related to India, but were not of Indian origin, if they lacked a link to a privacy policy, if they contained a broken link to a privacy policy, if the privacy policy was not in English, or if the privacy policy could not be copied for analysis. Privacy policies not written in English could not be included because structural differences in languages would make the readability statistics not comparable.

### Outcomes and Analyses

Apps were categorized according to whether they were interactive, noninteractive, or related to e-commerce, and then again categorized according to whether they were clinical, nonclinical, or related to e-commerce. (The same apps were placed in the e-commerce category under both categorizations.) Interactive apps were defined as apps that facilitate two-way discussions with a health expert (eg, doctors, therapists, nutritionists), apps which facilitate group chats, and apps with discussion forums. Apps involving interactions with supporting staff (eg, receptionists/customer care executives for online appointment booking) were categorized as noninteractive apps. A subset of the interactive apps was categorized as clinical if they involved interaction with a health expert; apps outside of the subset were categorized as nonclinical if they were not related to e-commerce.

Multiple metrics were used to evaluate the complexity of the app privacy policies: word count, sentences per paragraph, words per sentence, characters per word, average number of sentences per 100 words, average words with six or more characters, average number of sentences per 100 words, Flesch Reading Ease, Flesch-Kincaid Grade Level, Gunning Fog Score, SMOG Index, Coleman Liau Index, Automated Readability Index, Fry Grade Level, and Raygor Estimate Graph Grade Level. The mean, standard deviation, median, and interquartile range were calculated for each metric, separately for the diabetes and mental health apps. Metrics for diabetes apps and mental health apps were compared using *t* tests and Wilcoxon rank sum tests to assess for significant differences in mean and median, with *P*<.05 used as an indicator of significance. Chi-square tests were used to assess whether diabetes and mental health apps were similarly distributed between the interactive, noninteractive, and e-commerce categories, as well as between the clinical, nonclinical, and e-commerce categories, with *P*<.05 again used as an indicator of a significant association. All statistical analyses were conducted using STATA software version 13.

## Results

**Figure 1:**
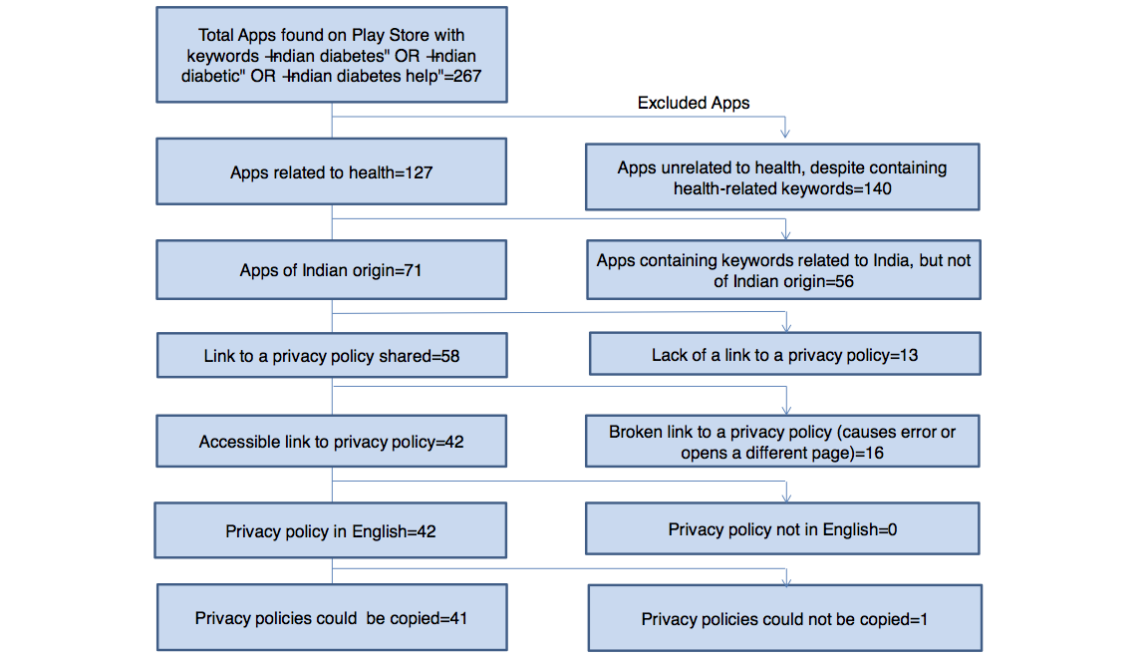
Selection of apps for diabetes.

**Figure 2:**
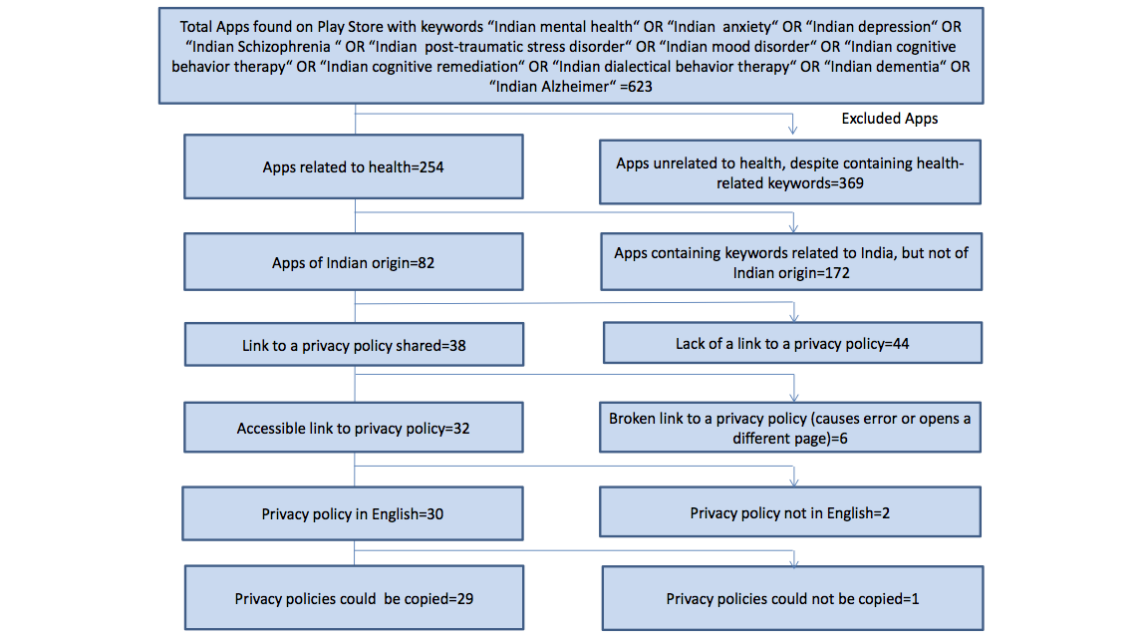
Selection of apps for mental health.

As is shown in Figure 1, a total of 267 potential Indian diabetes apps were found by searching the Google Play store. Of these apps, only 41 (15.4%) were included after the various exclusions were applied (nearly half the apps were unrelated to health despite containing health-related keywords). A similar process, shown in Figure 2, was applied to obtain the mental health app privacy policies. A total of 623 apps were returned by the initial searches of the Google Play store, but only 29 (4.7%) were included in the study after the exclusion criteria were applied. Of the total 70 apps included for analysis, eight apps (11%) were common for both diabetes and mental health.

The readability metrics for the app privacy are presented in Table 1. There were no significant differences in the readability of the privacy policies for apps for diabetes versus mental health. Similar results were found after excluding the common apps (n=8) from the analysis (data not shown). Overall, the metrics suggest that privacy policies may be difficult for people to read. A typical privacy policy is approximately as long as an article in an academic journal; a mean of 1875 words (SD 1448) for diabetes apps and 2421 words (SD 2102) for mental health apps. Although mental health app privacy policies had a higher mean and median word count, the difference was not statistically significant. Several of the metrics suggest that the privacy policies require a high degree of reading comprehension. The mean Flesch-Kincaid Grade Level for diabetes privacy policies was 13.9, and the mean for mental health privacy policies was 13.6. Furthermore, the 25th percentile of the interquartile range was 12.7 for diabetes apps and 12.4 for mental health apps. This suggests that understanding the majority of privacy policies requires reading at a college level. The Fry Grade Levels calculated—11.6 for diabetes apps and 12.4 for mental health apps—helps triangulate this finding. Although the mean Raygor Estimate Graph Grade Levels were lower (6.9 for diabetes and 7.4 for mental health), they also suggest that at least a middle school level of reading comprehension is required. In short, the privacy policies found in Indian apps for diabetes and mental health tend to be lengthy and difficult to read.

There were some differences in the nature of diabetes apps versus mental health apps, as shown in in Table 2. The vast majority of mental health apps (85%, 23/27) were interactive, whereas only a slight majority (23/39, 59%) of diabetes apps were interactive, a significant difference (*P*=.04). Mental health apps were likewise more likely to be clinical (82%) than diabetes apps (61%), although the distribution of apps between the clinical, nonclinical, and e-commerce categories was not significantly associated with app type (*P*=.06).

**Table 1:** Readability statistics of privacy policies of diabetes and mental health mobile apps (N=70).

Readability metric		Diabetes apps (n=41)		Mental health apps (n=29)		*P* value^a^	
**Word count**							
	Mean (SD)		1874.5 (1447.9)		2420.5 (2101.7)		.20
	Median (IQR^b^)		1520 (733-2278)		1783 (1117-3049)		.35
	Range		111-6010		163-9424		
**Sentences per paragraph**							
	Mean (SD)		2.9 (1.9)		2.5 (1.0)		.29
	Median (IQR)		2.6 (2.0-3.2)		2.2 (2.0-2.7)		.28
	Range		1.4-12.8		1.3-6.7		
**Words per sentence**							
	Mean (SD)		23.4 (4.9)		22.8 (5.2)		.60
	Median (IQR)		24.1 (21.4-26.9)		23.7 (20.6-25.2)		.40
	Range		11.7-32		8.5-37.5		
**Characters per word**							
	Mean (SD)		5.1 (0.2)		5.0 (0.2)		.47
	Median (IQR)		5.1 (5.0-5.2)		5.1 (5.0-5.1)		.54
	Range		4.6-5.5		4.8-5.3		
**Average number of sentences per 100 words^c^**							
	Mean (SD)		5.9 (5.1)		4.8 (0.9)		.27
	Median (IQR)		5 (4.2-5.8)		4.7 (4.2-5.3)		.34
	Range		3.4-37		3.0-7.1		
**Average words with ≥6 characters^d^**							
	Mean (SD)		19.8 (2.5)		19.4 (2.5)		.52
	Median (IQR)		20 (18.0-22.0)		20 (17.5-21)		.62
	Range		14.0-26.0		14.0-23.0		
**Average number of sentences per 100 words^d^**							
	Mean (SD)		5.1 (1.3)		4.8 (0.9)		.33
	Median (IQR)		5.0 (4.2-5.8)		4.7 (4.2-5.3)		.52
	Range		3.4-7.7		3.0-7.1		
**Flesch Reading Ease**							
	Mean (SD)		35.1 (8.8)		37.1 (8.6)		.36
	Median (IQR)		36.5 (28.3-38.2)		37.3 (31.3-42.5)		.41
	Range		18.5-50.6		21.9-55.0		
**Flesch-Kincaid Grade Level**							
	Mean (SD)		13.9 (2.3)		13.6 (2.4)		.50
	Median (IQR)		14 (12.7-15.1)		14 (12.4-15.0)		.51
	Range		9.3-18		8.1-19		
**Gunning Fog Score**							
	Mean (SD)		15.2 (2.2)		15.4 (1.8)		.72
	Median (IQR)		15.0 (13.9-16.8)		15.3 (14.7-16.1)		.55
	Range		11.5-20		11.5-19-9		

**SMOG Index**							
	Mean (SD)		12.1 (1.7)		12.1 (1.3)		.90
	Median (IQR)		11.9 (10.7-13.1)		12.2 (11.7-12.5)		.66
	Range		9.6-15.4		9.5-14.8		
**Coleman Liau Index**							
	Mean (SD)		14.2 (0.8)		13.9 (0.9)		.18
	Median (IQR)		14.5 (13.6-14.7)		14.2 (13.1-14.6)		.17
	Range		12.3-15.8		11.3-15.6		
**Automated Readability Index**							
	Mean (SD)		13.4 (2.8)		13.5 (2.2)		.86
	Median (IQR)		13.3 (11.5-14.9)		13.3 (12.8-14.6)		.85
	Range		8.8-18.4		8.5-18.6		
**Fry Grade Level**							
	Mean (SD)		11.6 (3.1)		12.4 (1.5)		.20
	Median (IQR)		12,0 (11.0-14.0)		13.0 (12.0-14.0)		.36
	Range		5.0-15.0		10.0-15.0		
**Raygor Estimate Graph Grade Level**							
	Mean (SD)		6.9 (1.1)		7.4 (2.2)		.25
	Median (IQR)		7.0 (6.0-8.0)		7.0 (7.0-8.0)		.70
	Range		4.0-9.0		4.0-18.0		

^a^*P* values are from using *t* tests of significance for the means and Wilcoxon rank sum tests for the medians.

^b^IQR: interquartile range.

^c^Fry word statistics.

^d^Raygor estimate word statistics.

**Table 2:** Characteristics of diabetes and mental health mobile apps by Indian developers.

Strata		Diabetes apps, n (%)	Mental health apps, n (%)	*P* value^a^
**Interactivity**				.04
	Interactive	23 (59.0)	23 (85.2)	
	Noninteractive	6 (15.4)	3 (11.1)	
	E-commerce	10 (25.6)	1 (3.7)	
**Use case**				.06
	Clinical	25 (61.0)	23 (82.1)	
	Nonclinical	6 (14.6)	4 (14.3)	
	E-commerce	10 (24.4)	1 (3.6)	

^a^*P* values are from chi-square tests of significance.

## Discussion

### Principal Findings

Although readability measures could be applied to any English-language privacy policies, there are a number of factors that make India a robust country to study. Because India is home to the world’s second-largest population of English speakers (after the United States) [23], the world’s second-largest base of mobile phone users (after China) [24], and legally enforces privacy rights [1], it has a well-developed market for English-based mHealth apps containing privacy policies. Given that the 2011 Census found that only 6% of the Indian population has a college education [25], whereas 30% of United States adults have a college education [26], the impact of privacy policies written at a college level is even more acute in India than in the United States. Furthermore, there is a great need for mHealth apps in India due to limited access to care in some parts of the country [27].

Lengthy, complex app privacy policies are not as likely to be read and understood as short, simple privacy policies. Depending on the metric used, the majority of app privacy policies evaluated may require a college education to comprehend. These findings are consistent with the prior finding by other researchers, who determined that the average grade level of the privacy policy of a mobile health app is grade 16 [15]. As mobile phones become more affordable to people of all incomes, the problems posed by complex privacy policies will likely intensify. In 2016, 25% of Indians had a mobile phone [28]. Among urban Indian mobile phone owners, the proportion who were less educated and earned a low income expanded from 38% to 45% between 2013 and 2015 [29].

Many other materials presented to the Indian public online are not this complex. Prior researchers have measured the Flesch-Kincaid Grade Level of a number of different websites administered by the Indian government and found their grade levels to be more moderate. For instance, the website of the Indian Air Force was written at an 8.4 grade level, whereas the website of the High Court of Bombay was written at a 6.6 grade level [30]. Although government-oriented websites may be inherently less complex than privacy policies, they do demonstrate that it is possible to convey information to the masses in a simple format.

Privacy policies incomprehensible to the majority of users are unfair because they do not allow users to make an informed choice between their desire for privacy and their desire to sacrifice some privacy to obtain the benefits of the app. As the Flesch-Kincaid Grade Level metrics suggest that some college education may be required to understand the privacy policies of three-quarters of apps, the majority of Indian diabetes and mental health app users are left with the choice between not using the majority of apps or agreeing to privacy policies that they may not fully understand. The potential for unfairness was highlighted when the provider of public Wi-Fi services, Purple, created a deliberately unreasonable privacy policy, requiring users to perform 1000 hours of community service on agreement and offering anyone who read to the end a prize if they contacted them. Of the 22,000 people who agreed to the policy, only one person contacted the company after having thoroughly read it [31].

### Recommendations

Privacy policies do not need to be incomprehensible. Complex concepts can be explained graphically to make them more accessible to people with limited reading comprehension. For instance, Creative Commons has created a standardized set of logos that indicate the rights that the authors of media have reserved [32]. These logos can be understood at a glance by people informed of their meaning. A similar approach could be applied to privacy policies if a standardized set of policies, with associated logos, were created.

Furthermore, standardized licenses like the GNU General Public License enable users to avoid the hassle of re-reading a long document each time they agree to use software by providing consistency across licenses [33]. Although users with lower levels of reading comprehension may not be able to understand standardized licenses, they too can benefit because more educated users have thoroughly vetted the policies to ensure that they are fair. When nonstandard policies are used, the likelihood of them being read by anyone (regardless of ability) is lower than when standardized policies are used. Furthermore, abstract concepts, such as deidentification and anonymization, can be explained with graphical representations so that they may be more widely understood. Finally, outreach efforts to help educate and explain the risk and benefits of digital technologies such as apps may be necessary to ensure individuals are equipped to make informed decisions regarding use. Already, online resources for digital technology ethics and privacy are emerging, such as the free-to-access and use Connected and Open Research Ethics Initiative [34].

### Limitations

The results of this study reflect two categories of health apps, from one country, from one app store, examined at one period in time. It is possible that the findings from this study are not generalizable to other types of apps, to other app stores (eg, the iTunes App Store), or to apps that are not of Indian origin. It is also possible that the privacy policies of apps may evolve over time. Furthermore, although apps addressing a broad selection of mental illnesses were analyzed, only apps addressing a single physical illness (diabetes) was analyzed. It was necessary to analyze apps addressing multiple mental illnesses rather than a single mental illness due to the relative paucity of apps addressing each illness. Even after this accommodation, the sample of apps related to mental health was substantially smaller than the sample of diabetes apps. The findings of this study may have been impacted by the set of keywords used during the app sample selection process. The 2017 actions of the Indian Supreme Court [1], which occurred after the data collection for this study was complete, may cause India-based app developers to evaluate whether their privacy policies remain consistent with the needs of Indian users and with Indian law.

### Conclusions

Although no differences were found between the complexity of the privacy policies of Indian apps for diabetes versus mental health, both were found to be complex. Some of the measures calculated suggested that a college level of reading comprehension is required to understand the typical privacy policy in an Indian app for diabetes or mental health. In order to ensure that the majority of India’s citizens are able to willfully consent to privacy policies when using apps, an effort to simplify privacy policies is needed.

## Abbreviations

DALY: disability-adjusted life years
